# Mechanical/Thermomechanical–Electromagnetic Multifunctional Cellulose Nanofibril-MXene Aerogel-Based Metamaterials

**DOI:** 10.34133/research.0900

**Published:** 2025-10-10

**Authors:** Kangkang Zhang, Chenyang Fan, Yanbo Wang, Lin Liu, Xian Wang, Chunwang Yang, Ning Li, Buapan Puangsin, Jun Li, Teerasak E-kobon, Jian Qiu, Yushan Yang

**Affiliations:** ^1^ College of Material and Chemical Engineering, Southwest Forestry University, Kunming 650224, Yunnan, People’s Republic of China.; ^2^ Department of Forest Products, Faculty of Forestry, Kasetsart University, Bangkok 10900, Thailand.; ^3^ Department of Genetics, Faculty of Science, Kasetsart University, Bangkok 10900, Thailand.

## Abstract

As the latest breakthrough in the field of metamaterials, the interfacial coupling of topological and multistable states of wooden metamaterials remarkably enhances functionality and enables transformative applications. However, the high coupling and complex geometrical properties of the microlattice may impose constrain on design flexibility and scalability. Herein, we present a mechanically/thermomechanically electromagnetically multifunctional CFO-CNFene metamaterial featuring stochastically tunable aggregation-prone magnetic particles by in situ deposition of homogeneous magnetic CoFe_2_O_4_ nanoparticles through intermolecular interactions in a lightweight CNFene aerogel backbone. The resulting lightweight porous CFO-CNFene metamaterials exhibit remarkable characteristics, including temperature-invariant superelasticity and ultralow thermal conductivity (26.12 mW m^−1^ K^−1^), and, combined with the synergistic enhancement provided by the agglomerate-free double cross-linked ferromagnetic cobalt ferrite nanoparticles, result in comprehensive multifunctionalities, including rapid deformation, fire resistance, infrared thermal camouflage behavior, electromagnetic interference shielding efficiency (99.999%), and electromagnetic wave absorption efficiency (99.99%). This breakthrough supports the development of advanced electromagnetic stealth technologies for military and civilian applications, including radar stealth and radiation protection.

## Introduction

Multifunctional wood metamaterials, although still relatively uncommon, are essential across various fields such as infrared thermal stealth, electromagnetic interference (EMI) shielding of military weaponry, and the thermal protection of electronic equipment [1–3]. The inability to provide effective camouflage technology for sources of electromagnetic wave (EMW) radiation, including on-board radar and electronic countermeasure equipment of military aircraft, can severely impair operational effectiveness [4]. Additionally, the critical issue of infrared thermal stealth in military aircraft cannot be overlooked [5]. To date, a range of lightweight nanowood derivatives, classified as wood metamaterials, have been developed. These include mechanically anisotropic, adiabatic anisotropic, and thermo-mechanically anisotropic multistable lightweight wood metamaterials [6], along with optical cellulosic metamaterials possessing daytime radiation-cooling “green” properties [7,8], broadband microwave-absorbing natural wood metamaterials [9], and robust wood-inspired metamaterial catalysts [10]. These wooden metamaterials integrate multidisciplinary knowledge encompassing nanotechnology, molecular biology, interfacial chemistry, and physical modeling, demonstrating exceptional acoustic, optical, electrical, thermal, and magnetic functionalities. This enables unprecedented applications in architecture, energy, environmental protection, national defense, and aerospace. Furthermore, advanced processing techniques and design concepts not only retain the inherent advantages of wood but also enhance functional properties such as ultrahigh strength, durability, and unique environmental adaptability [6]. Nevertheless, the complex microlattice geometrical properties of these materials introduce a high level of coupling and interdependencies, and restrict design flexibility and regulation. To realize controllable construction of multifunctional wooden metamaterials with multistable architectures, a thorough understanding of the constraints imposed by high coupling between structural and material properties, as well as issues related to nonlinearities, is required—particularly in the domains of mechanical/thermomechanical–electromagnetic multifunctionality. Therefore, the advancement of new multifunctional wooden metamaterials that surpass conventional chemical [11,12], thermal [13], acoustic [14], and optical metamaterials [15] could substantially contribute to scientific research and hold broad application prospects in essential sectors such as national health, defense, security, ocean exploration, and communications.

Wood, as a natural composite material, is abundant and renowned for its high strength-to-weight ratio, sound absorption, shock reduction, and biocompatibility. Throughout history, its research and application have consistently closely aligned with human endeavors, making it a long-standing area of study [16]. Its renewability and biodegradability further enhance its attractiveness [17]. Recently, mechanical/thermodynamic multistable lightweight nanowood derivatives have garnered increased attention, representing a subclass of wooden metamaterials [6]. This metamaterial combines mechanical anisotropy, adiabatic anisotropy, and thermomechanical anisotropy in order to achieve a balance between mechanical strength and thermal insulation performance. For instance, Douglas fir exhibits a staggered microlattice and a bimodal pore distribution of its fibers, reflecting a structural model that enables upright tree growth [10]. Nevertheless, prior research has uncovered limitations inherent in mechanical metamaterials, as their deformation configuration cannot be maintained once external mechanical loads or stimulus are removed. The distinct multistable configuration of mechanical metamaterials overcomes these limitations by preserving their initial configuration post-external load or stimuli, which leads to noteworthy mechanical properties such as negative compressibility, negative Poisson’s ratio, negative thermal expansion, and shear modulus blanking superfluid [17–19]. The emergence of mechanical/thermodynamic multistable lightweight nanowood derivatives exemplifies this new class of wooden metamaterial [6]. The high scientific research value of these materials paves the way for promising applications [14], alongside developments in energy absorption, logical operations, elastic wave control, and soft robotics [20–22]. However, significant challenges remain in achieving dual multistable controllable design and geometric regulation regarding the structural strength and functionality of wooden metamaterials. This includes the fine-tuning of supramolecular scales within hierarchical architectures, addressing cellular structural coupling among components, and facilitating rapid large-scale industrial development.

Wooden metamaterials, characterized as oriented porous framework materials, cleverly merge the self-healing capabilities of cellulose nanofibrils (CNFs) present in wood fiber skeleton with microscale mechanical constraints [6]. As a sustainable functional material, wooden metamaterials exhibit superior mechanical, thermal, and acoustic characteristics owing to their unique structural design and nanotechnological regulation, offering exciting potentials in construction, energy, and environmental protection. Furthermore, anisotropic nanocellulose aerogels derived from nonbrittle precursors may mitigate existing limitations [23]. The structural design and optimization of multistable wood-based metamaterials face a core challenge: achieving dynamic control over material composition, gradients, interfaces, microstructure, and morphology while enabling precise prediction of their responses under complex operating conditions. Consequently, anisotropic nanowood and wood aerogels have surfaced, demonstrating excellent thermal insulation capabilities and substantial promise in thermal management [24]. Specifically, balsa wood achieves remarkably high strength-to-weight and stiffness-to-weight ratios in the longitudinal direction of the cell wall [25]. Recent research has highlighted ultralight lattices and foam structures valued for their outstanding specific stiffness, specific strength, and impact resistance [26]. Nature excels at engineering robust, lightweight, layered, multifunctional materials derived from cellular microstructures formed throughout evolution, often surpassing artificial materials in unique material characteristics—some of which are rare or even nonexistent in nature, such as zero/negative Poisson’s ratio [27]. Notable examples include honeycomb wood (e.g., balsa and cork), foamy trabecular bone, and plant parenchyma [28]. Additionally, cyclically arranged nanofibril aggregates in nanowood facilitate anisotropic heat flow due to their high aspect ratio, effectively obstructing heat channels and contributing to exceptional outstanding thermal insulation [16]. Nevertheless, substantial plastic deformation poses a challenge [23]. While obstacles related to preparation processes, performance stability, and cost control persist, ongoing research and technological advancements suggest that wooden metamaterials are poised for significant progress in the future development of sustainable materials. However, the intricate relationship between structure and performance complicates the fulfillment of diverse and evolving practical needs.

Herein, we present mechanical/thermomechanical–electromagnetic multifunctional CoFe_2_O_4_-CNF-MXene (CFO-CNFene) metamaterials, synthesized from lightweight CNF-MXene (CNFene) aerogels that serve as templates for the agglomeration-free dual cross-linked ferromagnetic cobalt ferrite nanoparticles. The CNFene hybrid 3-dimensional (3D) framework, achieved though intermolecular interactions and fabricated using noncontact freeze-drying techniques, features a dual cross-linked architecture that enhances both electronic and ionic conductivity as well as mechanical strength. Additionally, it synergistically boosts magnetic, microwave absorption, and electromagnetic shielding capabilities due to its integrated multilevel interface architecture, which comprises various microscale and nanoscale components. The design approach effectively utilizes a lightweight porous CNFene aerogel skeleton, enabling the random deposition of homogeneous magnetic CoFe_2_O_4_ nanoparticles and addressing the aggregation issue of high-concentration magnetic nanoparticles, resulting in mechanical/thermomechanical–electromagnetic multifunctional CFO-CNFene metamaterials. The properties of as-prepared CFO-CNFene metamaterials include lightweight structure, superhydrophobicity, complete recovery from significant deformation, temperature-invariant superelasticity, low thermal conductivity, fire resistance, infrared thermal camouflage behavior, EMI shielding, and EMW absorption performance. These remarkable properties stem from the synergistic effects of the multiscale micro-nanoarchitecture of 3D CNFene hybrid framework and the agglomeration-free dual cross-linked ferromagnetic cobalt ferrite nanoparticles.

## Results and Discussion

### Synthesis of CFO-CNFene metamaterials

**Fig. 1.**
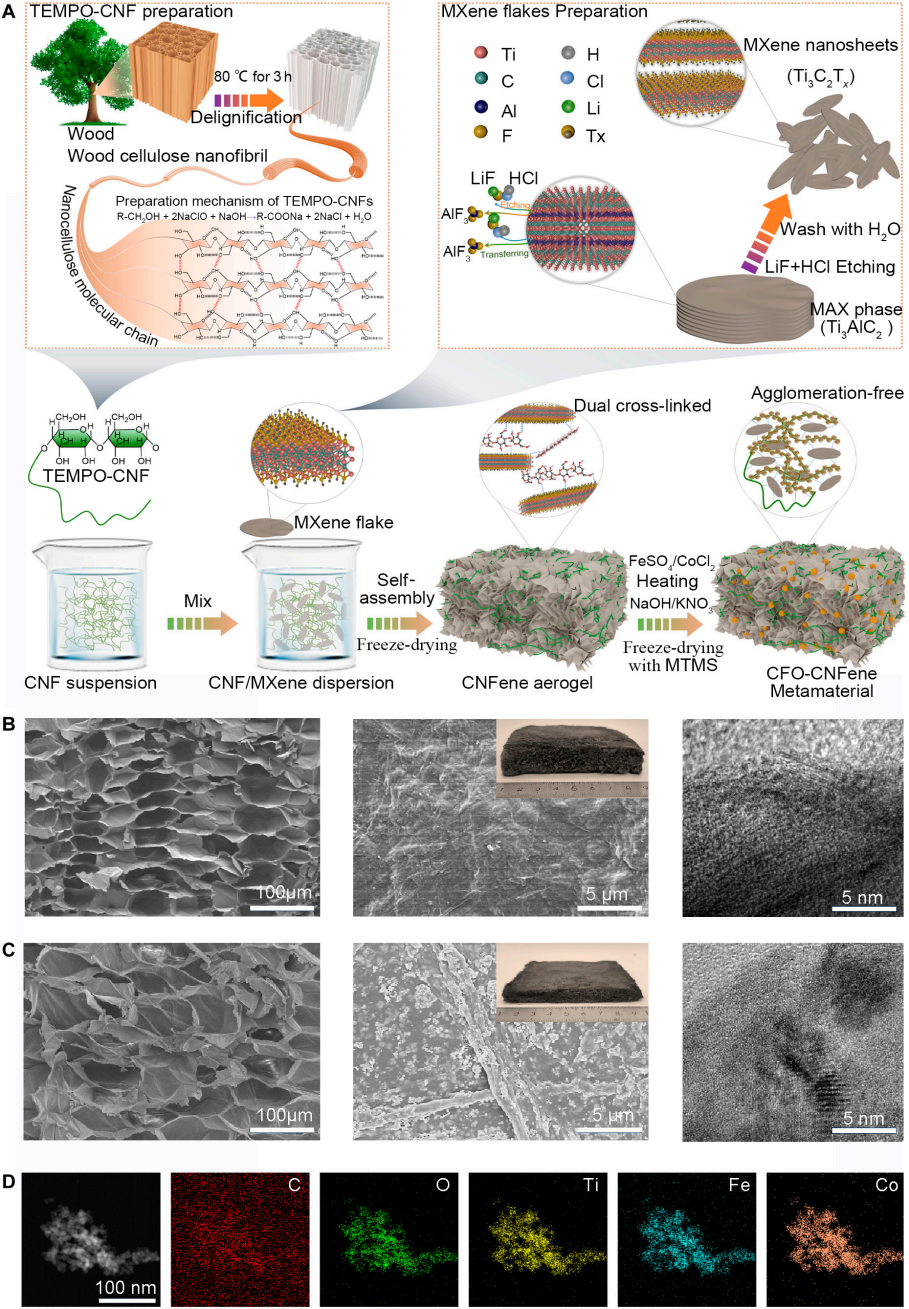
Development and structural characterization of CFO-CNFene metamaterials. (A) Manufacturing process of CFO-CNFene metamaterials. (B) Architectural characteristics of CNFene aerogels. From left to right: SEM image, optical image, and HRTEM image. (C) Architectural characteristics of CFO-CNFene metamaterials. From left to right: SEM image, optical image, and HRTEM image. (D) HAADF image of CFO-CNFene metamaterials and corresponding elemental distributions of C, O, Ti, Fe, and Co.

The functional elements of wood cellulose were investigated through 2 key aspects: nano-controllable topological order and functional directional recasting. The primary processes include the directional incorporations of exogenous functional nanoparticles, precise optimization and control of pore architecture, and the design and adjustment of interface topological deformation configurations. We propose a method for preparing mechanical multistable lightweight nanofiber metamaterials based on the concept of “functional element + order”. Initially, 2,2,6,6-tetramethylpiperidin-1-oxyl (TEMPO)-oxidized cellulose nanofibrils (TEMPO-CNFs) and Ti_3_C_2_T*_x_* MXene nanosheets were synthesized separately, as shown in Fig. 1A and Fig. S1. An aqueous dispersion of Ti_3_C_2_T*_x_* MXene nanosheets was obtained from a precisely manipulated, dense Ti_3_AlC_2_ MAX precursor. The preparation of CFO-CNFene metamaterials entailed 3 steps: (a) synthesizing TEMPO-CNF, (b) synthesizing Ti_3_C_2_T*_x_* MXene nanosheets, and (c) developing CNFene aerogels as mechanical–electromagnetic multifunctional metamaterials from lightweight CNFene aerogels serving as templates for the agglomeration-free dual cross-linked ferromagnetic cobalt ferrite nanoparticles (Fig. S2).

Initially, a chemical purification treatment combined with mechanical nanofibrillation was employed. Following a top-down approach, functional element fibrils units of TEMPO-CNF were prepared from the nanoscale architectures of purified wood fiber skeleton [29]. Specifically, TEMPO was utilized to oxidize the purified wood fiber skeleton into carboxylic acid fiber. Under magnetic stirring, a cellulose aqueous suspension with a mass fraction of 0.5% was formed, and the solution’s pH was subsequently adjusted to 10, repeating until the pH of the mixed suspension fell below 0.5. Excess chemicals in the cellulose suspension were removed through centrifugation and filtration, immediately replacing the wastewater with deionized water. This purification process was repeated multiple times to acquire pure TEMPO-CNF. The centrifuged TEMPO-CNFs were then mixed with water and subjected to ultrafine nanodissociation using an ultrasonic cell crusher, yielding transparent ultrafine wood cellulose units referred to as wood cellulose functional elements. Concurrently, multilayer Ti_3_C_2_T*_x_* MXene particles and delaminated Ti_3_C_2_T*_x_* MXene nanosheets were obtained from Ti_3_AlC_2_ MAX phase through a modified minimally intensive layer delamination method [30].

The TEMPO-CNF flocculation suspension underwent ultrasonically accelerated dispersion in an ice-water bath. Subsequently, the Ti_3_C_2_T*_x_* MXene nanosheets were uniformly mixed into the suspension, which was then contactlessly freeze-dried to produce the CNFene aerogel. By employing a mixed solvent as an innovative reaction medium, we utilized a sol-gel method in conjunction with in situ growth to create mechanical–electromagnetic multifunctional metamaterials from lightweight CNFene aerogel serving as templates for the agglomeration-free dual cross-linked ferromagnetic cobalt ferrite nanoparticles. A crucial step involved immersing the CNFene aerogel in a FeSO_4_/CoCl_2_ aqueous solution for thermal precipitation of nonmagnetic metal hydroxides/oxides. The precipitation precursor was then converted into ferrite crystal nanoparticles using a NaOH/KNO_3_ mixed solution, leading to magnetic aerogels capable of enduring significant deformations. These magnetic CoFe_2_O_4_ nanoparticles demonstrated strong saturation magnetization, facilitating effective EMI shielding, EMW absorption, and various magnetism-related applications. Ultimately, contactless freeze-drying in a methyltrimethoxysilane (MTMS) environment produced mechanical/thermomechanical–electromagnetic multifunctional CFO-CNFene metamaterials. As shown in Fig. 1B, the developed CNFene aerogels exhibit a uniform and regular porous architecture, with pore sizes ranging from several hundred micrometers to several millimeters, as observed in the cross-sectional scanning electron microscopy (SEM) image. This pore architecture supports the penetration of polymer monomers or solutions into the aerogels for further functionalization and mitigates capillary force that would otherwise lead to aerogel shrinkage. Interestingly, while pure MXene aerogels and CNF aerogels possess continuous 3D porous architectures, they fail to maintain their original configuration upon immersion in solutions, thus hindering their modification and the encapsulation with functional magnetic CoFe_2_O_4_ nanoparticles, even for CNFene aerogels. This limitation may stem from the strong hydrogen bonding interactions established by TEMPO-CNF embedded within the Ti_3_C_2_T*_x_* MXene nanosheets.

As shown in Fig. 1C, the porous CNFene aerogels derived from TEMPO-CNF@MXene suspension using contactless freeze-drying processes serve as templates for the nonaggregate in situ deposition of ferromagnetic cobalt ferrite nanoparticles. The morphological images of the CFO-CNFene metamaterials skeleton network reveal that the homogeneous CoFe_2_O_4_ nanoparticles are densely packed within the 3D mesoporous architecture, minimizing van der Waals interaction and facilitating the nonaggregate growth of ferromagnetic cobalt ferrite nanoparticles. The porous structural components of CNFene aerogels and ferromagnetic cobalt ferrite nanoparticles were modified in situ through hydrothermal deposition, resulting in a stable 3D skeleton network during the growth process. Following directional contactless freeze-drying, the abundant hydroxyl and carboxyl groups on the surface of TEMPO-CNF facilitated hydrogen bonding interactions with Ti_3_C_2_T*_x_* MXene nanosheets and magnetic CoFe_2_O_4_ nanoparticles, thereby fostering the formation of the CFO-CNFene prepolymer gel skeleton. Concurrently, unidirectional ice crystals applied a repulsive force on TEMPO-CNF, Ti_3_C_2_T*_x_* MXene nanosheets, and magnetic CoFe_2_O_4_ nanoparticles, pushing them away from the front of the prepolymer gel and leading to the development of a distinct honeycomb porous microstructure. This microstructure features cell walls aligned in the direction of ice crystal growth. To elucidate the distribution of internal components, representative transmission electron microscopy (TEM) images of the internal architecture and the corresponding elemental distribution were employed for characterization. The results, as shown in Fig. 1D, reveal a relatively uniform distribution of C, O, Ti, Fe, and Co elements across the entire fiber surface. The micro–nano binary architecture of the CFO-CNFene metamaterials, which arose from the magnetic CoFe_2_O_4_ nanoparticles, can be further elucidated through Ostwald ripening. The presence of magnetic CoFe_2_O_4_ nanoparticles on the surface of the lightweight porous CFO-CNFene metamaterials can be interpreted as a uniform micro–nano binary oxide architecture, which is consistent with the microstructural data obtained from high-resolution TEM (HRTEM) image and the accompanying elemental analysis presented in Fig. 1D.

### Material characterization of CFO-CNFene metamaterials

**Fig. 2.**
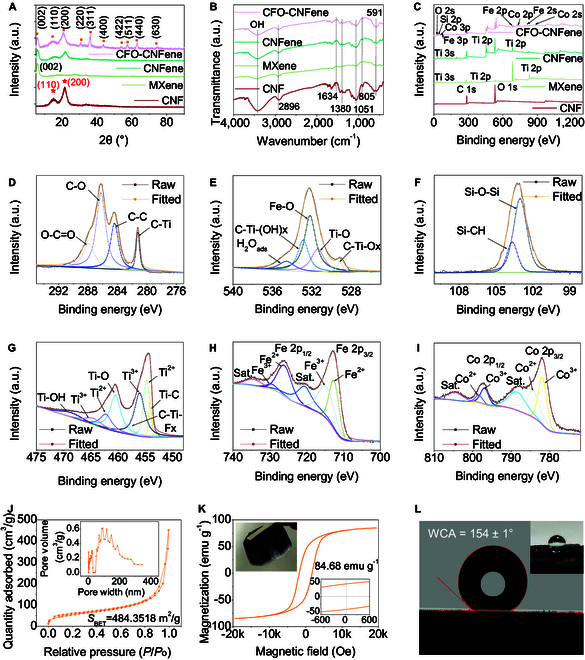
Material characterization of CFO-CNFene metamaterials. (A) XRD patterns of CNF aerogel, CNFene aerogel, and CFO-CNFene metamaterial. (B) FTIR spectra of CNF aerogel, CNFene aerogel, and CFO-CNFene metamaterial. (C) XPS spectra of CNF aerogel, CNFene aerogel, and CFO-CNFene metamaterial. (D) C 1s spectra of CFO-CNFene metamaterial. (E) O 1s spectra of CFO-CNFene metamaterial. (F) Si 2p spectra of CFO-CNFene metamaterial. (G) Ti 2p spectra of CFO-CNFene metamaterial. (H) Fe 2p spectra of CFO-CNFene metamaterial. (I) Co 2p spectra of CFO-CNFene metamaterial. (J) N_2_ sorption isotherms and gap widths distribution of CFO-CNFene metamaterial. (K) Magnetic hysteresis loops of CFO-CNFene metamaterial. (L) Experimental snapshot of superhydrophobic CFO-CNFene metamaterial.

**Fig. 3.**
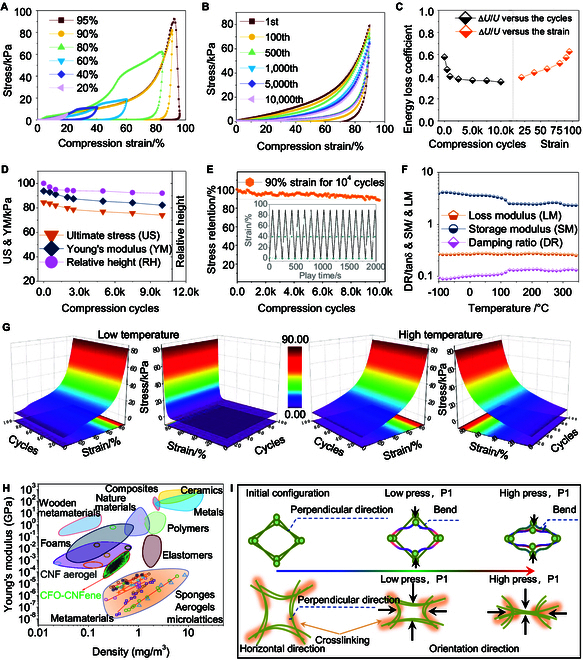
Mechanical/thermomechanical properties characterization of CFO-CNFene metamaterials. (A) Uniaxial compression of CFO-CNFene metamaterials with strain up to 95%. (B) The compressive stress–strain curves of CFO-CNFene metamaterial at 90% strain for 10,000 cycles. (C) Mechanical energy loss coefficient of CFO-CNFene metamaterial. (D) Ultimate stress (US), Young’s modulus (YM), and relative height (RH) of CFO-CNFene metamaterial. (E) Stress retention of CFO-CNFene metamaterial. (F) Storage modulus, loss modulus, and damping ratio of CFO-CNFene metamaterial. (G) Temperature-invariant superelasticity of CFO-CNFene metamaterial. (H) Ashby plot of Young’s modulus versus density of CFO-CNFene metamaterials compared with other lightweight aerogels. (I) A schematic illustration of the framework deformation.

The x-ray diffraction (XRD) results for CNF aerogel, CNFene aerogel, and CFO-CNFene metamaterial are shown in Fig. 2A. The diffraction peaks at 15.0° and 21.6° in the XRD spectrum of CFO-CNFene metamaterial correspond to the characteristic crystal planes (101) and (002) of the TEMPO-CNF unit [31]. A distinct peak at 6.7° in the XRD spectrum indicates the (002) peak of Ti_3_C_2_T*_x_* MXene nanosheets. Furthermore, the peaks at 18.33°, 30.0°, 36.3°, 43.9°, 54.2°, 57.5°, and 62.8° in the XRD spectra shift to the right, corresponding to the characteristic diffraction peaks of magnetic CoFe_2_O_4_ nanoparticles (111), (220), (311), (222), (400), (440), and (630) (JCPDF#22-1086), indicating a reduction in interlayer gap size and a strong interaction between Ti_3_C_2_T*_x_* MXene nanosheets and TEMPO-CNF [32]. Fourier transform infrared spectroscopy (FTIR) (Fig. 2B) and x-ray photoelectron spectroscopy (XPS) (Fig. 3C to I) were employed to study the cross-linking behavior between TEMPO-CNF, Ti_3_C_2_T*_x_* MXene nanosheets, and magnetic CoFe_2_O_4_ nanoparticles within CFO-CNFene metamaterial. The FTIR spectrum of CNFene aerogel verifies the formation of hydrogen bonds between Ti_3_C_2_T_x_ MXene nanosheets and TEMPO-CNF molecular chains. Distinct hydroxyl peaks (3,250 to 3,450 cm^−1^) in the FTIR spectra of CFO-CNFene metamaterial further affirm the presence of strong hydrogen bonding interactions among component nanoparticles. Additionally, the absorption peak of 591 and 410 cm^−1^, resulting from Fe-O or Co-O vibration, substantiates that the hydroxyl/CoFe_2_O_4_ nanoparticle complex can be adsorbed onto the surface of the CNFene aerogel structural unit through proton exchange reactions with the hydroxyl groups found within the TEMPO-CNF molecular chains.

The Ti-O-C band (459.3 eV) in the XPS Ti 2p spectrum of CFO-CNFene metamaterial (Fig. 2C) indicates covalent bonding between Ti_3_C_2_T*_x_* MXene nanosheets and EMPO-CNF, as well as interactions between Ti_3_C_2_T*_x_* MXene nanosheets and magnetic CoFe_2_O_4_ nanoparticles. The presence of C, O, Si, Ti, Fe, and Co elements within CFO-CNFene metamaterial confirms the successful integration of magnetic CoFe_2_O_4_ nanoparticles and CNFene aerogel. High-resolution C 1s scanning XPS spectroscopy was performed to gain insights into the atomic bonding present within CFO-CNFene metamaterial. As shown in Fig. 2D, the high-resolution C1s XPS spectrum of CFO-CNFene metamaterial displays peaks at 287.9, 286.3, 284.4, and 281.2 eV, corresponding to the binding energies of O-C=O, C-O, C-C/C-H, and C-Ti bonds within the porous architecture, respectively. The high-resolution O 1s curve reveals 5 peaks at 534.7, 532.9, 532.1, 531.4, and 529.2 eV (Fig. 2E), which are associated with H_2_O_ads_, C-Ti-(OH)*_x_*, Fe-O, Ti-O, and C-Ti-O*_x_*, respectively. The Si 2p spectrum presents 2 peaks at 1,033.1 and 103.7 eV (Fig. 2F), along with a peak at 102.9 eV attributable to the Si-O bond resulting from MTMS. The Ti 2p, Fe 2p, and Co 2p spectra in CFO-CNFene metamaterial were comprehensively characterized (Fig. 2G). The observed Ti-Ti-Fx peak originates from the Ti_3_C_2_T*_x_* MXene nanosheets, while the Ti-O peak may result from minor oxidation of TiO_2_ during the preparation or hybridization of Ti_3_C_2_T*_x_* MXene nanosheets [30]. The +2 and +3 valence states of Fe and Co in CFO-CNFene metamaterial are evidenced (Fig. 2H and I).

To comprehensively assess the porosity of CFO-CNFene metamaterial, N_2_ adsorption–desorption isotherm was acquired through Brunauer–Emmett–Teller (BET)-specific surface area testing, yielding a specific surface area of 484.35 m^2^ g^−1^ (Fig. 2J). Notably, CFO-CNFene metamaterials exhibit rich macropore and mesopore configuration, with average gap sizes ranging from tens of nanometers to hundreds of nanometers. In Fig. 2K**,** room-temperature hysteresis lines for CFO-CNFene metamaterials demonstrate an S-shape, indicating ferromagnetic characteristics with saturation magnetization strength reaching 84.68 emu g^−1^. The inset illustrates the macro magnetism of CFO-CNFene metamaterials; specifically, a neodymium magnet can easily pick up a specimen with a volume 10 times larger than its own without significant saturation magnetization strength. This result is primarily attributed to the deposition of magnetic CoFe_2_O_4_ nanoparticles, which display favorable crystallinity modifying the surface of the CNFene aerogel. Additionally, since the final contactless freeze-drying process was performed in an MTMS atmosphere, which utilized the CNFene aerogel backbone of the CFO-CNFene metamaterials and the hydrophilic groups present in the magnetic CoFe_2_O_4_ nanoparticles, chemical grafting modification imparts hydrophobicity. As shown in Fig. 2L, water droplets on the surface of CFO-CNFene metamaterials are spherical, maintaining a water contact angle of up to 154° ± 1° and thereby indicating excellent superhydrophobicity. This water resistance enables usability in high humidity or wet conditions.

### Mechanical/thermomechanical properties characterization of CFO-CNFene metamaterials

To evaluate the mechanical properties of CFO-CNFene metamaterials under uniaxial quasi-static compressive strains, cyclic compression tests were firstly conducted on CNF aerogel, CNFene aerogel, and CFO-CNFene metamaterials at 80% strain. The stress–strain curves exhibited an initial linear elastic behavior, deviating to present a near plateau stress deformation, as stress decreased more rapidly with slight hysteresis observed during unloading. The underlying mechanisms for this hysteresis may include intra- and inter-wall van der Waals adhesion and friction during deformation, as well as materials’ ability to revert to their initial conformation upon unloading. Moreover, CFO-CNFene metamaterials were capable of returning to their initial state following pressure release when the compressive strain was augmented from 20% to 95% (Fig. 3A). Following the continuous increase to 95% and beyond, the modulus of elasticity increased to 100.15 kPa, but at this point, it is assumed that part of the internal configuration that has collapsed at this level contributed to reduced resilience. Subsequently, CFO-CNFene metamaterial was subjected to cyclic compression at a significant strain of 90% for 10,000 cycles (Fig. 3B), revealing that the specimen conformation degraded by approximately 5%, yet stress retention remained above 85%, exemplifying excellent fatigue resistance. Following such high strain cyclic compression, stable energy loss coefficients were observed. These coefficients decreased from the 1st cycle (∆*U*/*U* = 0.46) to 0.35 at the 10,000th cycle, indicating that the structural unit of CFO-CNFene metamaterial could effectively function as an energy damping metamaterial (Fig. 3C). The Young’s modulus, ultimate stress, and relative height of the CFO-CNFene metamaterial were quantified (Fig. 3D), demonstrating that major stress recession primarily occurred during the initial compression cycle. The relative height was maintained at less than 1% residual strain through consecutive cycles, as the configuration tended to stabilize, evidencing stress retention above 85% (Fig. 3E) and denoting excellent compressive resilience of CFO-CNFene metamaterials.

To investigate the temperature-invariant superelasticity of CFO-CNFene metamaterials, their thermal stability was evaluated using dynamic mechanical analysis (DMA) over a temperature range of −100 to 350 °C (Fig. 3F), establishing that their viscoelasticity remained nearly unchanged throughout this temperature range due to suppressed hardening and degradation rates of the fibrils in CFO-CNFene metamaterial. Following this evaluation, the CFO-CNFene metamaterial underwent large strain rebound cycling tests at near 0 °C and a hot bench at near 500 °C. The colored 3D surfaces of CFO-CNFene metamaterial under compressive stress–strains demonstrated exceptional fatigue resistance and near-zero plastic deformation after 1 to 100 compression cycles (Fig. 3G). The results indicated that stress degradation following 100 cycles at 80% strain was 11.14% and 13.25%, respectively, thereby confirming the outstanding temperature-invariant superelasticity. Furthermore, through intermolecular interactions, CFO-CNFene metamaterial was synergistically enhanced using micro- and nanoscale architectures modified by nanoparticle hybridization, resulting in newly developed CFO-CNFene metamaterial with porous frameworks constituted exclusively by aligned high-strength porous frameworks and CNFene scaffolds. The specific strength of CFO-CNFene metamaterial was found to be comparable with many lignocellulosic aerogel composites of similar types, other lightweight aerogels, and cellulose nanofoams based on sponge structures (Fig. 3H) [6,24,33,34]. This phenomenon is primarily ascribed to the bending and tensile dominant features of fundamental elastic units within its 3D multi-walled porous honeycomb skeleton, enabling the compressed CFO-CNFene metamaterials to revert to their original configuration following large-scale geometrical deformation (Fig. 3I). This behavior can likewise be expressed through Maxwell’s equations to delineate conditions for tensile dominant behavior [35], where tensile-dominant configuration provides enhanced stiffness and strength in comparison to bending-dominant configuration, thereby improving the load-bearing paths in pore walls and amplifying the stiffness of the continuous unoriented pore structure, which ultimately enhances the stability and fatigue resistance of CFO-CNFene metamaterials.

### Thermal insulation properties of CFO-CNFene metamaterials

**Fig. 4.**
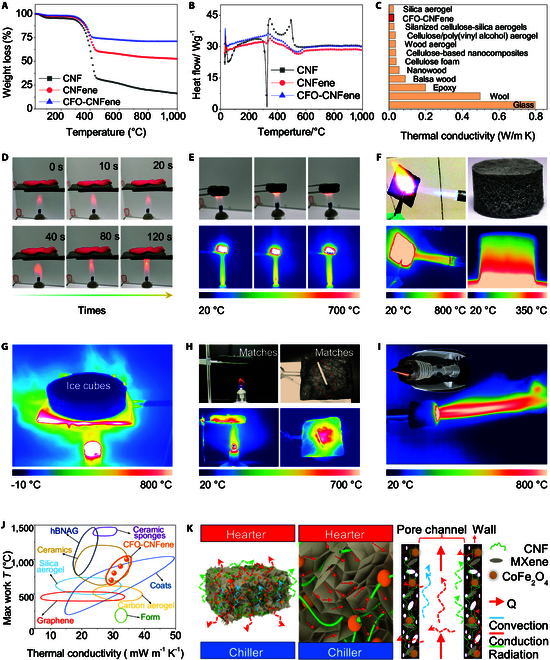
Thermal insulation properties of CFO-CNFene metamaterials. (A) Thermogravimetric curves of CNF aerogel, CNFene aerogel, and CFO-CNFene metamaterial. (B) Differential scanning calorimeter curves of CNF aerogel, CNFene aerogel, and CFO-CNFene metamaterial. (C) Comparison of thermal conductivity of CFO-CNFene metamaterial with those of other materials. (D) Experimental snapshot showcasing thermal protection of fresh flowers under an alcohol lamp using CFO-CNFene metamaterial. (E) Experimental snapshot demonstrating the flame-retardant properties of CFO-CNFene metamaterial. (F) Characterization of flame-retardant and thermal insulation properties of CFO-CNFene metamaterial. (G) Infrared pseudo-color image of CFO-CNFene metamaterial for thermal protection of ice cubes under a butane blowtorch. (H) Experimental snapshot demonstrating excellent thermal superinsulation capabilities of CFO-CNFene metamaterial for matches. (I) Infrared pseudo-color image of a butane nozzle protected by CFO-CNFene metamaterial. (J) Comparison of thermal conductivity and maximum operating temperature of CFO-CNFene metamaterial with other aerogels. (K) Schematic illustration of the flame-retardant and heat insulation mechanism of CFO-CNFene metamaterial.

To investigate the thermal stability of CFO-CNFene metamaterials, evaluations were conducted using a combined thermogravimetric-differential scanning calorimetry (TG-DSC) analysis, as shown in Fig. 4A and B. The results indicate that CNFene aerogel and CFO-CNFene metamaterial exhibit superior thermal stability compared to CNF aerogel; however, the presence of dense magnetic CoFe_2_O_4_ nanoparticles on the surface of CFO-CNFene metamaterial enhances thermal stability, enabling the retention of over 75% of residual weight at 1,000 °C (Fig. 4A). This mass loss of approximately 25% primarily results from the dissipation of water on the specimen surface (about 3% to 5%) and depolymerization pyrolysis of CNF (about 20%), with a maximum thermal decomposition temperature of about 330 °C. Additionally, observations from the DSC curves (Fig. 4B) indicate that the weight loss rate of CFO-CNFene metamaterial is lower than that of CNF aerogel and CNFene aerogel during the heating process. However, when the temperature exceeds approximately 380 °C, the weight loss rate of CFO-CNFene metamaterial and CNFene aerogel surpasses that of CNF aerogel. In contrast, the thermal properties of various specimens under air atmosphere, primarily due to the oxidation (combustion) reactions, reveal that thermal effects within CFO-CNFene metamaterial are not pronounced, thus demonstrating excellent thermal stability.

Thermal conductivity tests reveal that CFO-CNFene metamaterial exhibit a thermal conductivity of 26.12 mW m^−1^ K^−1^, categorizing them among other lightweight thermal insulators (Fig. 4C) [6,29,36,37]. Remarkably, the unique porous configuration of CFO-CNFene metamaterial inhibits air conduction and convection, allowing for superior thermal insulation properties. Consequently, there was no observed wilting or carbonization of fresh petals after 120 s of high-temperature expose, with 5-mm-thick CFO-CNFene metamaterials placed at the midpoint between flowers and an alcohol lamp flame (Fig. 4D). In Fig. 4E, an experiment demonstrates that CFO-CNFene metamaterials exhibit no significant flame generation upon exposure to an alcohol lamp flame, showcasing notable flame-retardant self-extinguishing properties. In contrast, cellulose, polymers, and carbonaceous insulating foam architectures are prone to collapse or ignite under similar conditions. Figure 4F presents an experimental snapshot illustrating the exposed surface subjected to a butane torch flame at 1,000 °C and a heated plate surface at 350 °C, affirming the exceptional flame-resistant and thermal insulating properties of CFO-CNFene metamaterials. Further confirmation of these properties is derived from the tests involving ice cubes and flammable matches (Fig. 4G and H). Interestingly, 5-mm-thick CFO-CNFene metamaterial wrapped around a butane torch nozzle demonstrated minimal surface temperature increases, registering less than 100 °C after 30 min of heating, underscoring their remarkable high-temperature insulation capabilities. Overall, CFO-CNFene metamaterials exhibit high thermal stability, low thermal conductivity, and flame-retardant insulation properties, providing a foundation for sustainable thermal superinsulation protective materials under complex temperature conditions. Figure 4I highlights the advantages of CFO-CNFene metamaterial for flame-retardant insulation applications by comparing the performance of existing insulators such as silica aerogels, carbon aerogels, ceramic aerogels, ceramic sponges, and foams [33,34]. The result illustrates that CFO-CNFene metamaterials retain significant advantages across the measured parameters.

Figure 4J presents a schematic representation of the heat transfer mechanism applicable to the 3 forms of heat transfer (radiation, convection, and conduction) in lightweight porous CFO-CNFene metamaterials. Considering that the mechanical properties of CFO-CNFene metamaterials resemble those of foams, the thermal convection within these foams must also be factored into calculating overall thermal conductivity. Specifically, total thermal conductivity is expressed as λ_T_ = λ_s_ + λ_g_ + λ_c_ + λ_r_. Given that the voids in CFO-CNFene metamaterial are comparable to the mean free path of air molecules (N_2_ and O_2_), molecular collisions within these voids are suppressed, thus achieving the Knudsen effect and leading to an ultralow λ_g_ value. Furthermore, air movement is highly restricted, inhibiting convective conduction [6,29]. Additionally, the infinite-length pathway provided by the pore architecture of CFO-CNFene metamaterial facilitates effective reflection and absorption of thermal radiation. Moreover, the interface connections between adjacent pores (Fig. 4K) creates additional thermal resistance, with the contribution of thermal radiation to λ being substantially lower than that of thermal convection and conduction within typical operating temperature range for thermal insulation. Consequently, the conduction influence on heat transfer in CFO-CNFene metamaterials is more complex compared to radiation and convection, dramatically reducing conductivity and enhancing thermal insulation capabilities.

### Infrared thermal camouflage behavior of CFO-CNFene metamaterials

**Fig. 5.**
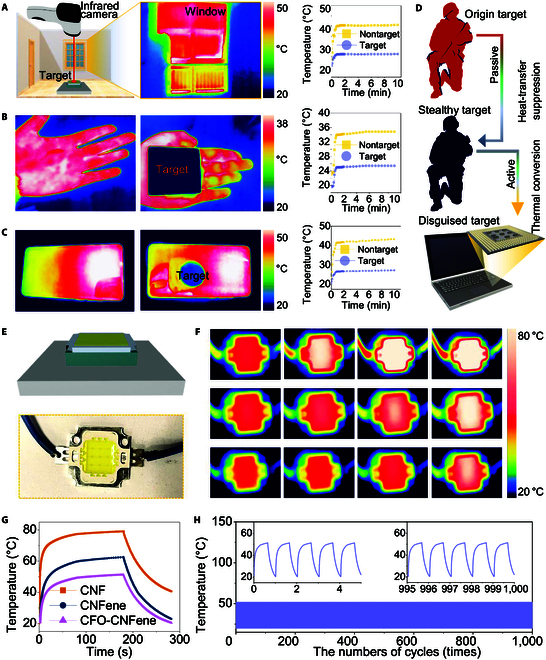
Infrared thermal camouflage behavior of CFO-CNFene metamaterials. (A) Passive isolation of building interiors from cold or hot outdoors. (B) Infrared thermal camouflage of CFO-CNFene metamaterial for human body. (C) Infrared thermal camouflage of CFO-CNFene metamaterial for electronic devices. (D) Schematic illustration of the active and passive thermal camouflage of CFO-CNFene metamaterial. (E) Schematic illustration of thermal management of LED chips utilizing integrated TIM and heat sinks. (F) Infrared pseudo-color image of LED module. (G) Temperature profiles of LED chips utilizing different heat sinks, including CNF aerogel, CNFene aerogel, and CFO-CNFene metamaterial. (H) Thermal shock stability of CFO-CNFene metamaterial as heat sink during cyclic heating and cooling.

Infrared stealth technology refers to measures aimed at attenuating the infrared signature of aircraft in critical detection vectors targeted by infrared measurement devices. Reducing the range and intensity of infrared radiation detected is essential for enhancing aircraft survival by minimizing the detection likelihood and tracking accuracy of infrared-seeking missiles. Contemporary thermal infrared camouflage technologies are more inclined toward hardware design for aircraft and military electronics, with the intent of achieving thermal infrared stealth capabilities for weaponry and electronic systems. Prolonged localized hotspots may adversely affect sensitive components, risking irreversible safety hazards and property security. While flame-retardant insulation materials can mitigate the radiant temperature of hot objects, CFO-CNFene metamaterials have the potential to serve as thermal camouflage materials. To assess the thermal camouflage behavior of CFO-CNFene metamaterials, an infrared camera was implemented to evaluate their effectiveness. Results in Fig. 5A illustrate that CFO-CNFene metamaterials can passively isolate the interior of a building from cold or hot external temperatures, maintaining an indoor temperature that is a relatively comfortable temperature—showcasing exceptional thermal insulation and energy saving. Figure 5B and C depict the practical application of CFO-CNFene metamaterial in infrared thermal camouflage for the human body and electronic devices. When CFO-CNFene metamaterial is applied over a hand at a low temperature of 20 °C, the infrared pseudo-color map indicates that the temperature of the covered area is comparable to that of the environment, visually representing successfully concealment of the body heat signature (Fig. 5B). This property also translates to mobile devices, as evidenced by the simulated infrared measurement of the area concealed using CNFene metamaterials appearing similar to the surrounding environment (Fig. 5C). In contrast, the uncovered portion of the thermal infrared pseudo-color map reveals medium to high temperatures that are markedly higher than those of the covered area, consistent with thermal variations observed on the device’s surface. The stark contrast observed in the thermal infrared pseudo-color maps confirms the capability of CFO-CNFene metamaterials to isolate or blend the thermal signature of objects, thus evading detection by infrared thermal imaging systems.

To elaborate on the capacity for infrared thermal camouflage, Fig. 5D illustrates a schematic allowing CFO-CNFene metamaterials to function in diverse environmental conditions. In low-temperature environments, CFO-CNFene metamaterials serve as insulating layers that cover high-temperature targets, effectively isolating their thermal characteristics. This passive infrared thermal stealth behavior allows the high-temperature target to seamlessly blend with their surroundings, rendering them invisible to infrared imaging systems. Conversely, in high-temperature environments, low temperature adorned with CFO-CNFene metamaterial can camouflage against cooler objects due to the excellent thermal insulation. This enables their surface temperature to actively heat synchronize with the ambient conditions, allowing them to blend seamlessly with colder objects. Throughout this process, the infrared thermal camouflage properties of CFO-CNFene metamaterial successfully obscure target temperatures, either rendering them inconspicuous or allowing them to disappear into the background environment, generating false thermal distribution signals that can mislead the adversary. This capability enhances the efficacy of stealth and deception mechanisms.

Furthermore, CFO-CNFene metamaterials were employed as heat sinks for the thermal interface of LED chips (Fig. 5E), and thermal infrared cameras recorded temperatures of the light-emitting diode (LED) chips, visually illustrating real thermal management capabilities through thermal infrared pseudo-color mapping. To evaluate the cooling efficiency of CNFene metamaterial in the operation context of LED chips, CNF aerogel, CNFene aerogel, and CFO-CNFene metamaterial were employed as thermal interface materials between the LED lamps and copper heat sinks, respectively. The electrical readout indicated that the surface temperature of LED chips utilizing CFO-CNFene metamaterials as heat sinks stabilized around 50 °C after 180 s of activation, aligning with the practical application requirements of LED chips. Additionally, an infrared camera recorded surface temperature variations of the LED lamps as a function of lighting time (Fig. 5F). Upon activation, LED surface temperatures exhibited rapid increases that gradually stabilized over time. It is noteworthy that surface temperatures of LED lamps using CNFene aerogel and CFO-CNFene metamaterial as heat sinks not only increased at a slower rate but also remained lower than the equilibrium temperature of LED units using CNF aerogel (80.7 °C). Upon switching off the light, surface temperature rapidly returned to ambient temperature levels, demonstrating highly efficient and reversible light-to-heat conversion capabilities (Fig. 5G). Moreover, the thermal shock stability of LED lamps utilizing CFO-CNFene metamaterial as heat sinks was assessed through repeated heating and cooling shock experiments, revealing consistent temperature profiles that remained stable at approximately 51.4 °C after 100 cycles (Fig. 5H). This result highlights the reliable heat dissipation capability of CFO-CNFene metamaterials, emphasizing their advantages as thermal interface materials in thermal management applications to improve efficiency and prolong LED lamp longevity. The infrared thermal camouflage capability of CFO-CNFene metamaterial was found to be comparable with many aerogel composites of similar types, other lightweight hierarchical metamaterials, rGO hybrid aerogels, and polypyrrole foams based on hierarchically porous configuration [38–40].

### EMI shielding and EMW absorption performance of CFO-CNFene metamaterials

**Fig. 6.**
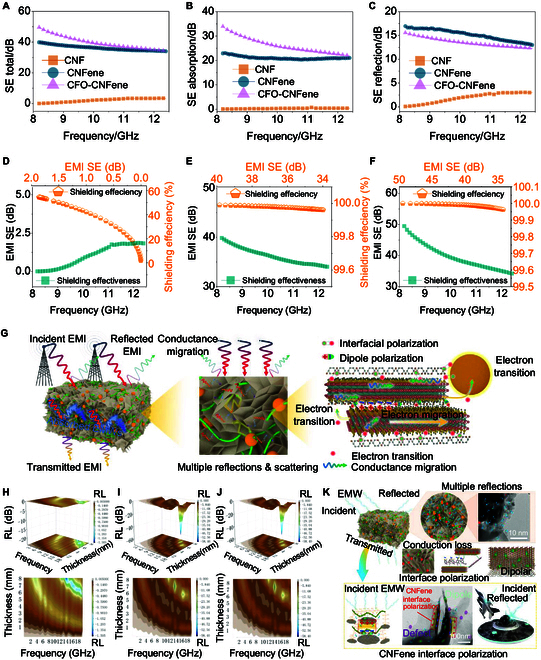
EMI shielding performances of CFO-CNFene metamaterials. (A) EMI SE_total_ of CNF aerogel, CNFene aerogel, and CFO-CNFene metamaterial. (B) EMI SE_absorption_ of CNF aerogel, CNFene aerogel, and CFO-CNFene metamaterial. (C) EMI SE_reflection_ of CNF aerogel, CNFene aerogel, and CFO-CNFene metamaterial. (D) Shielding effectiveness and shielding efficiency of CNF aerogel. (E) Shielding effectiveness and shielding efficiency of CNFene aerogel. (F) Shielding effectiveness and shielding efficiency of CFO-CNFene metamaterial. (G) Schematic illustration of the EMI shielding mechanism of CFO-CNFene metamaterial. (H) 3D and 2D reflection loss contour plots for CNF aerogel. (I) 3D and 2D reflection loss contour plots for CNFene aerogel. (J) 3D and 2D reflection loss contour plots for CFO-CNFene metamaterial. (K) Schematic illustrations of EMW absorption mechanisms for CFO-CNFene metamaterial.

In addition to the excellent mechanical/thermomechanical–electromagnetic properties, CFO-CNFene metamaterials inherit the high electrical conductivity and ultrahigh EMI shielding effectiveness (SSE/t). As shown in Fig. 6A, CFO-CNFene metamaterials with a thickness of approximately 3 mm exhibit exceptional EMI shielding effectiveness (SE) across the frequency range of 8 to 12 GHz. Overall EMI SE was assessed by calculating reflectance and absorptance (Fig. 6B and C). The results reveal that EMI shielding in CFO-CNFene metamaterials is predominantly governed by absorption efficiency, attributed to the incident EMWs being reflected back and forth within the CFO-CNFene metamaterials, consequently creating an infinite transmission path that continuously absorbs EMWs. A detailed assessment of the shielding efficiency and SE of CNF aerogel, CNFene aerogel, and CFO-CNFene metamaterial was presented in Fig. 6D to F, with CFO-CNFene metamaterial achieving the highest EMI shielding value of 52.4 dB, capable of blocking 99.999% of incident EMI. By evaluating EMI utilizing efficiency defined as EMI SE divided by the mass percentage of conductive filler, CFO-CNFene metamaterial achieved an outstanding utilization efficiency of 137.6 dB cm^2^ g^−1^, placing it among the highest values recorded [41–44]. It is evident that only CNFene aerogel and CFO-CNFene metamaterial possess EMI shielding capabilities, with the latter demonstrating the highest EMI SE. This enhanced performance is attributed to the presence of magnetic CoFe_2_O_4_ nanoparticles, which incorporate additional absorption-dominated shielding mechanism. Meanwhile, enhancing the reflection coefficient (*R*), absorption coefficient (*A*), and transmission coefficient (*T*) augmented the surface EMI shielding mechanism of CFO-CNFene metamaterial. As compared to CNF aerogel, CNFene aerogel, and CFO-CNFene metamaterial, higher *A* values were observed, attributed to the porous microstructure and multicomponent-rich interfacial reflection and absorption. By evaluating EMI utilizing efficiency defined as EMI SE divided by the mass percentage of conductive filler, CFO-CNFene metamaterial achieved an outstanding utilization efficiency of 137.6 dB cm^2^ g^−1^. Such unprecedented EMI SE can greatly facilitate the practical application of MXene in industrial settings through considerable reductions in operational costs. Figure 6G illustrates that the effective conductive network formed by the CNFene aerogel can facilitate multiple reflections at the interface while residual EMWs that penetrate the CNFene aerogel undergo further internal reflections and absorption between the porous architectures. This results in prolonged propagation paths of EMWs, promoting their complete attenuation. In addition, the abundant surface terminals and local defects present in the Ti_3_C_2_T*_x_* MXene nanosheets generate dipole polarization, while magnetic CoFe_2_O_4_ nanoparticles help regulate the excessive conductivity and optimize impedance matching conditions, thereby enhancing the EMI shielding capability of CFO-CNFene metamaterials.

Figure S3 displays the reflection loss values of CNF aerogel, CNFene aerogel, and CFO-CNFene metamaterial. The results indicate that the reflection loss value of CNF aerogel is below 2.5 dB, rendering it negligible, while the reflection loss values for CNFene aerogel and CFO-CNFene metamaterial approach 60 dB, with CFO-CNFene metamaterial performing notably better. Additionally, 3D and 2D reflection loss contour plots of CNF aerogel, CNFene aerogel, and CFO-CNFene metamaterial across an absorptive capacity range of 1 to 8 mm are presented in Fig. 6H to J. The results show that CNF aerogel, CNFene aerogel, and CFO-CNFene metamaterial exhibit respective reflection loss values of 1.69, 58.54, and 59.35 dB at frequencies of 13.37, 13.26, and 13.25 GHz, establishing them as a member of the group of microwave absorption solid materials [31,40]. This highlights that both CNFene aerogel and CFO-CNFene metamaterial possess commendable microwave absorption capabilities. The microwave absorption mechanism for CFO-CNFene metamaterial is shown in Fig. 6K. The 3D porous architecture of CFO-CNFene metamaterial, combined with the synergistic influences of ferromagnetic CoFe_2_O_4_ and conductive Ti_3_C_2_T*_x_* MXene nanosheets under varying electromagnetic fields, in addition to large aggregated charges and interactions between CoFe_2_O_4_ and Ti_3_C_2_T*_x_* MXene nanosheets at heterogeneous interfaces. Moreover, the resultant 3D porous network configuration, which features a high specific surface area, prolongs the propagation path of incident electromagnetic wave, manifesting further reflections and scattering between Ti_3_C_2_Tx MXene flakes and magnetic CoFe_2_O_4_ nanoparticles, thereby contributing to effective absorption and raising the relaxation loss peak in the absorber. Upon exposure to alternating electromagnetic fields, microcurrents are formed within this conduction network, leading to conduction losses and the conversion of electromagnetic energy into heat. CoFe_2_O_4_ also plays a critical role in the magnetic losses experienced by CFO-CNFene metamaterial through eddy current losses and natural resonance.

## Conclusion

In summary, we propose that the mechanical/thermomechanical–electromagnetic multifunctional CFO-CNFene metamaterials achieve uniform and in situ deposition of magnetic CoFe_2_O_4_ nanoparticles—with a tendency for aggregation deterioration—onto a lightweight CNFene aerogel framework through intermolecular interactions. This effectively results in agglomeration-free double cross-linking. Subsequently, the resultant materials exhibit a porosity. CFO-CNFene metamaterials not only showcase low density and thermal conductivity but also facilitate rapid recovery to their original morphology following substantial deformation (up to 95% strain) under complex environmental conditions, thus displaying outstanding temperature-invariant superelasticity. Additionally, the synergistic effects of the multiscale micro–nano architecture in the CNFene hybrid framework and the agglomeration-free double cross-linked ferromagnetic cobalt ferrite nanoparticles collectively enhance properties such as fire resistance, infrared thermal camouflage, EMI shielding, and EMW absorption performance. This multifaceted capability presents a feasible approach for developing advanced electromagnetic stealth radar systems and military equipment, paving the way for applications in infrared radar stealth, EMI protection, electromagnetic radiation safeguards, and other sectors related to defense and civil technologies.

## Materials and Methods

### Materials

Benzene (AR, ≥99.5%), anhydrous ethanol (AR, water ≤0.3%), sodium chlorite (NaClO_2_, AR, 80%), glacial acetic acid (AR, 36%), potassium hydroxide (KOH, AR, 85%), sodium hydroxide (NaOH, AR, 96%), hydrochloric acid (HCl, 36% to 38%), TEMPO (98%), lithium fluoride (LiF, AR, 99.99%), commercially pure titanium aluminum carbide MAX phase powder (Ti_3_AlC_2_, 98%, 200 mesh), iron sulfate heptahydrate (FeSO_4_, ≥99.95%), cobalt(II) chloride (CoCl_2_, ≥97%), and potassium nitrate (KNO_3_, ≥99%) were purchased from Shanghai Aladin Chemical Technology Co., Ltd.

### Preparation of repurified wood fiber skeleton

Six grams of wood block was placed in a Soxhlet extractor and refluxed for 6 h at 90 °C using a benzene–ethanol mixture (volume ratio of 1:1), followed by air-drying. The benzene–alcohol solution was refluxed and recovered. The air-dried sample was then transferred into a 250-ml conical flask. Subsequently, 200 ml of a 5% (w/w) NaClO_2_ solution, buffered with glacial acetic acid to a pH of 4.5, was added. The flask was sealed with plastic wrap, shaken well, and heated in a 75 °C water bath for 1 h. Following this, 1.5 ml of glacial acetic acid and 1.9899 g of sodium chlorite were added. The mixture was shaken well and heated in a 75 °C water bath for 1 h. This step was repeated 5 to 6 times until the sample turned white. The mixture was then cooled to room temperature and washed with copious amounts of water until neutral. The wet holocellulose skeleton was placed in a 3-necked flask and treated with 100 ml of a 2 wt% KOH solution at 90 °C for 2 h, followed by repeated washing with deionized water. The sample was bleached again with sodium chlorite at 75 °C for 1 h, and subsequently treated with KOH at 90 °C for 2 h. The final residue was transferred to a conical flask and treated with 100 ml of a 1 wt% HCl solution at 80 °C for 2 h, followed by repeated washing with copious amounts of water until neutral, yielding the purified wood fiber skeleton.

### Fabrication of ultrafine TEMPO-CNFs

Initially, TEMPO was employed to oxidize delignified wood chips or pulps into carboxylic fibers, generating a cellulose aqueous suspension with a mass fraction of 0.5%. Following 15 min of magnetic stirring, the pH of the resulting solution was readjusted to 10, repeating the procedure until the mixed suspension’s pH fell below 0.5. Centrifugation at 9,000 rpm for 30 min removed excess chemicals from the cellulose suspension. Subsequently, deionized water replaced the waste liquid, repeating this purification process several times to ensure the elimination of residual chemicals, yielding purified oxidized cellulose. The purified TEMPO-oxidized cellulose was combined with water or ethanol and treated with an ultrasonic cell crusher (JY98-IIIDN, Ningbo Xinzhi Biotechnology Co., Ltd.) for 60 min (with a power output of 900 W and a working cycle set to 50%), resulting in ultrafine CNFs, called TEMPO-CNFs.

### Fabrication of Ti_3_C_2_T*_x_* MXene nanosheets

Commercially pure Ti_3_AlC_2_ MAX phase material is preferred for etching accordion-shaped Ti_3_C_2_T*_x_* MXene nanosheets. Multilayer Ti_3_C_2_T*_x_* particles and corresponding delaminated Ti_3_C_2_T*_x_* MXene nanosheets were peeled off from Ti_3_AlC_2_ MAX using a modified minimum intensity delamination (MILD) technique. Specifically, 1.6 g of LiF was gradually added to 20 ml of 9 M HCl and stirred for 5 min until dissolved; thereafter, 1 g of Ti_3_AlC_2_ MAX phase was slowly incorporated over 10 min to prevent boiling and sputtering, followed by continuous stirring at 35 to 45 °C for 24 h. The mixture was washed repeatedly with deionized water, centrifuging at a speed of 3,500 rpm for 6 to 8 times (each lasting 5 min), until the pH of the solution exceeded 6. The collected precipitate was combined with 100 ml of water and ultrasonically treated for 3 h in an argon-protection atmosphere, followed by centrifugation at 3,500 rpm for 1 h to extract the supernatant.

### Fabrication of CNFene aerogel

Initially, the TEMPO-CNF aqueous dispersion was mixed with the delaminated Ti_3_C_2_T*_x_* MXene nanosheet dispersion, stirred for 30 min using a magnetic stirrer, followed by ultrasonic treatment for 5 h (60 W). The mixed flocculated suspension was uniformly dispersed in an ice bath for 15 min at a specific weight ratio. Then, the mixed TEMPO-CNF@MXene flocculated precursor solution was freeze-dried at −42 °C for 24 h. Ultimately, CNFene aerogel was constructed by contactless freeze-drying (at −55 °C for 0.5 h and reduced pressure of approximately 10 μPa, maintained for 72 h).

### Fabrication of CFO-CNFene metamaterials

The dried CNFene aerogel template was first immersed in an aqueous FeSO_4_/CoCl_2_ solution at ambient temperature, followed by an in situ hydrothermal treatment at 90 °C for 3 h. It was then immersed in a 90 °C solution of NaOH/KNO_3_ to convert into ferrite crystalline nanoparticles. Finally, the cellulose-based magnetic aerogel was obtained in an MTMS environment by a contactless freeze-drying technique.

### Characterizations

Microstructural analysis was performed via field emission scanning electron microscopy (SU8010, Japan) and TEM images. Crystal structures were identified through XRD (BRUKER D8 ADVANCE) with a Johansson monochromator (Cu Kα1 radiation, λ = 1.5406 Å) at a scanning rate (2θ) of 4° min^−1^. FTIR was conducted on a Thermo Nicolet NEXUS-470 infrared spectrometer across the wavenumber range of 400 to 4,000 cm^−1^. XPS (Thermo ESCALAB 250 XI, Thermo Fisher Scientific K-Alpha, USA) was recorded in a vacuum environment (*P* < 10^−8^ bar) using a monochromatic K-Alpha source (1,486.6 eV). The BET method was used to calculate the BET-specific surface area and pore size distribution. Magnetic properties were assessed by a vibrating sample magnetometer (VSM, LakeShore 7404, USA) from Oxford Instruments under maximum applied magnetic field conditions. Superhydrophobicity was evaluated via an optical contact angle measurement system (Data Physics, Germany). All mechanical properties were determined using an electromechanical universal testing machine CMT 6104 at a load of 200 N and a strain rate of 10 mm min^−1^. Viscoelastic mechanical behavior was quantified by a dynamic thermomechanical analyzer (DMA, Mettler-Toledo, Switzerland) with STARe SW 16.00 at 5 Hz frequency. Thermal conductivity was measured by a Hot Disk thermal constant analyzer (Hot Disk TPS2500, Switzerland) by the transient plane source method. Temperature curves and thermal infrared pseudo-color images were recorded by an infrared thermometer GM1651 (−30 to 1,650 °C) and a FOTRIC348+ infrared thermal imager (−20 to 1,550 °C). EMI shielding performance was assessed using a Gironde vector network analyzer (VNA, PNA-N5244A, Agilent, USA) across the X-band (8 to 12 GHz, 22.9 × 10.2 × 2 mm^3^). The microwave parameters, complex magnetic permeability, and complex dielectric constant were measured using a network analyzer (Agilent E5071C, USA).

## Data Availability

All data are available in the main text or the Supplementary Materials.
