# Supplementary material for: Mechanical/Thermomechanical–Electromagnetic Multifunctional Cellulose Nanofibril-MXene Aerogel-Based Metamaterials
Source: Research (Wash D C). 2025 Oct 10;8:0900. doi: 10.34133/research.0900 (PMC12604525; doi:10.34133/research.0900)
Supplement: Supplementary 1 — Figs. S1 to S3 [file research.0900.f1.docx]

SUPPLEMENTARY MATERIALS

Mechanical/thermomechanical-electromagnetic multifunctional cellulose nanofibril-MXene aerogel-based metamaterials

Kangkang Zhang^1^, Chenyang Fan^1^, Yanbo Wang^1^, Lin Liu^1^, Xian Wang^1^, Chunwang Yang^1^, Ning Li^1^, Buapan Puangsin^2^, Jun Li^1*^, Teerasak E-kobon^3^, Jian Qiu^1*^, Yushan Yang^1*^

^1^ College of Material and Chemical Engineering, Southwest Forestry University, Kunming 650224, Yunnan, People’s Republic of China.

^2^ Department of Forest Products, Faculty of Forestry, Kasetsart University, Bangkok 10900, Thailand

^3^ Department of Genetics, Faculty of Science, Kasetsart University, Bangkok 10900, Thailand.

*Corresponding author: E-mail addresses: [lijun@swfu.edu.cn (J Li)](mailto:lijun@swfu.edu.cn%20(J%20Li)); [qiujianswfu@163.com (J Qiu)](mailto:qiujianswfu@163.com%20(J%20Qiu)); [ysyoung@swfu.edu.cn (Y Yang)](mailto:ysyoung@swfu.edu.cn%20(Y%20Yang))


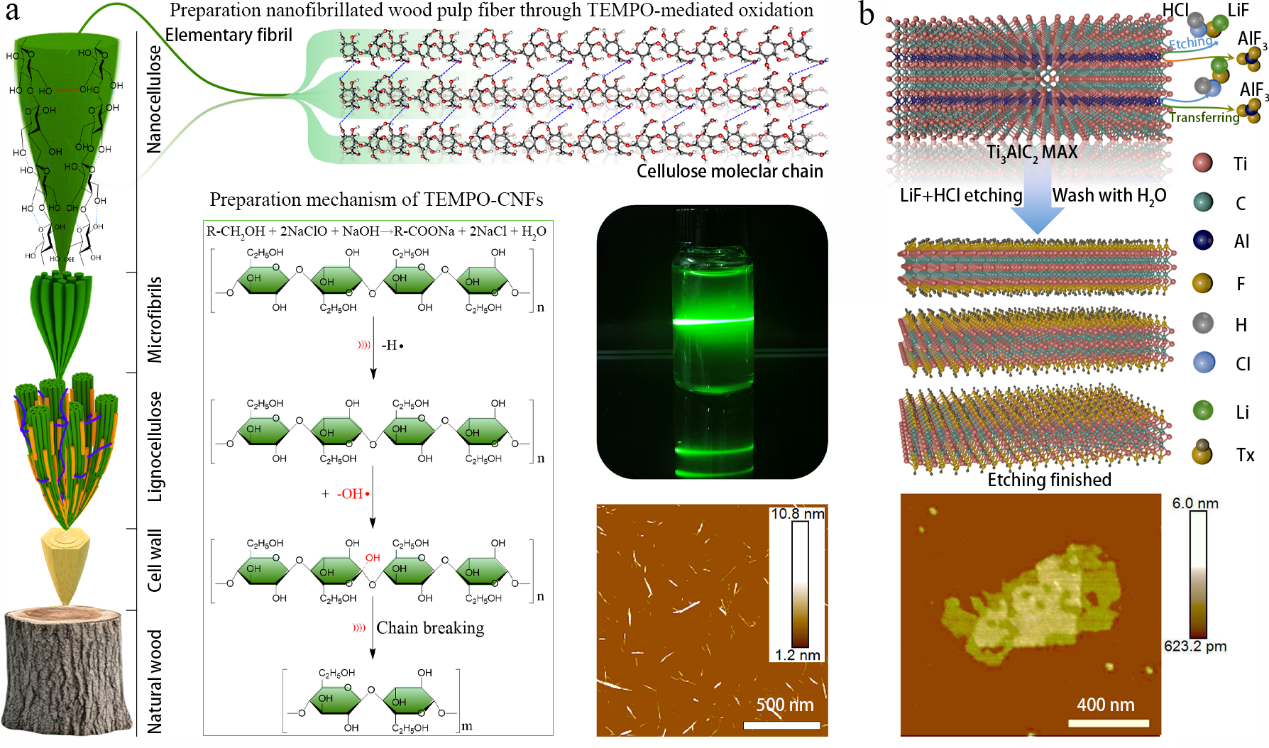


**Figure S 1** Fabrication of TEMPO-mediated wood cellulose nanofibril-and MXene flakes.


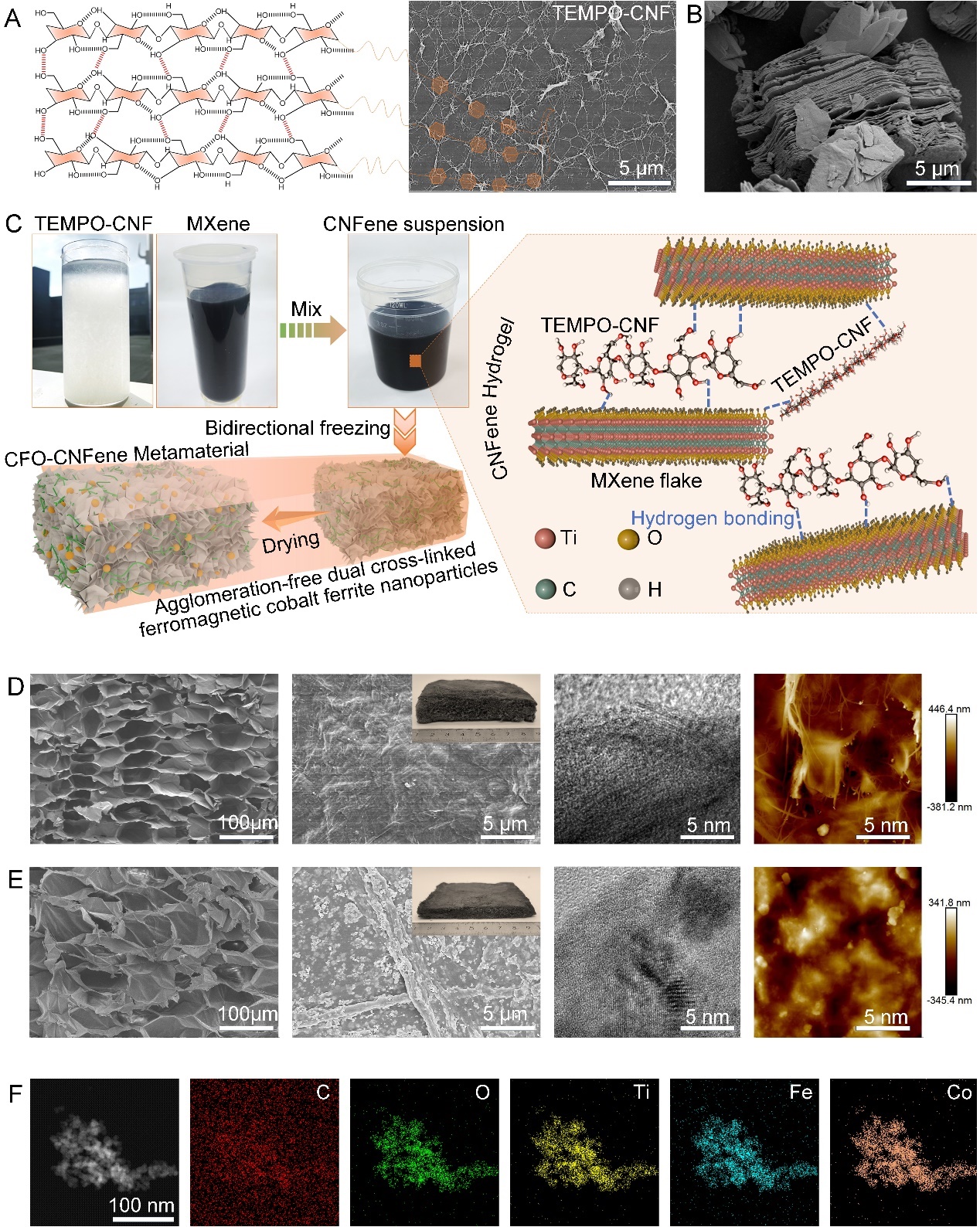


**Figure S 2** **Structural characterization of** **CFO-CNFene metamaterials.** **(A)** SEM image of TEMPO-CNF. (**B**) SEM image of Ti_3_C_2_Tx MXene flakes. (**C**) Schematic illustrating the fabrication of CNFene aerogels and CFO-CNFene metamaterials.


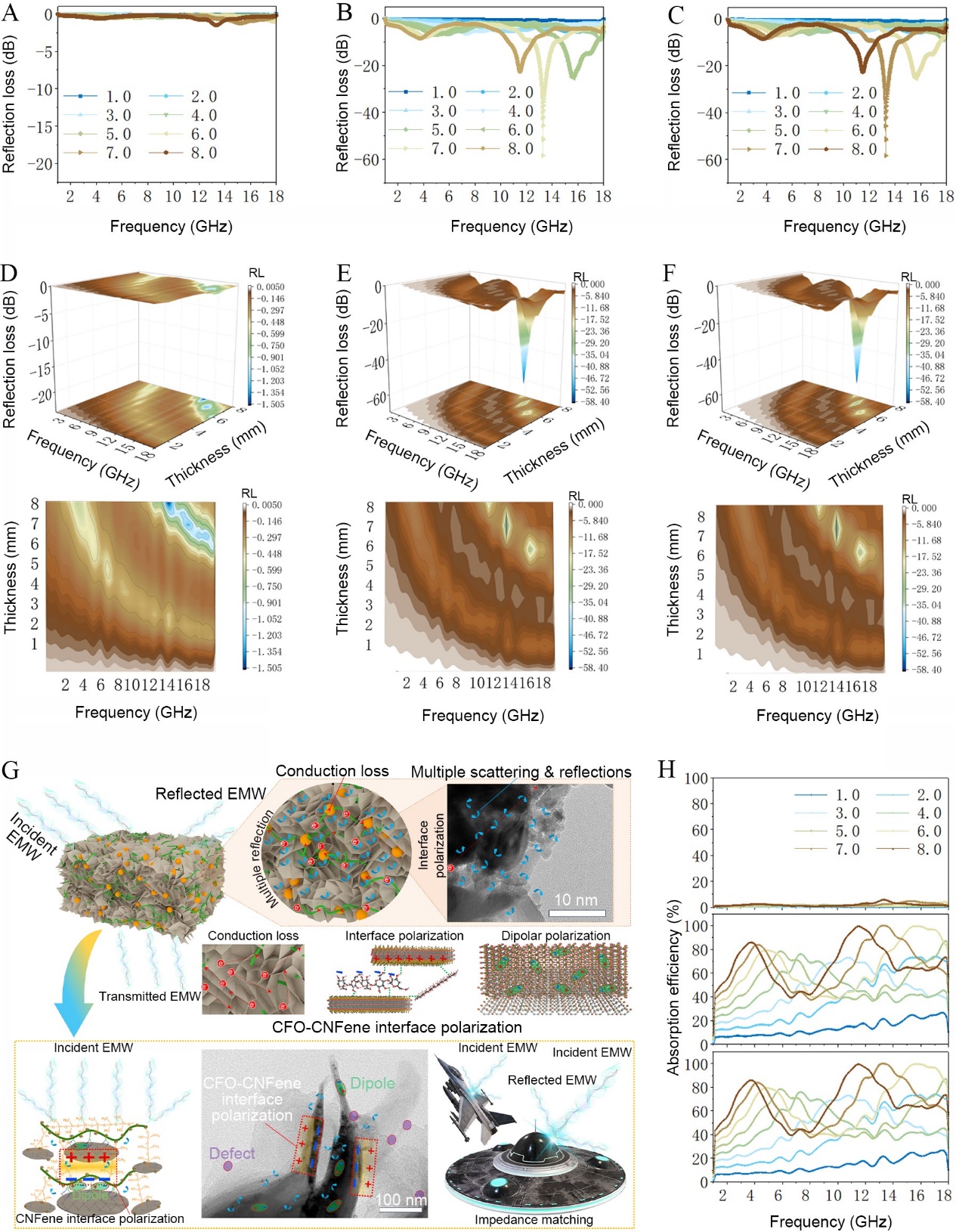


**Figure S 3 EMW absorption performance of CFO-CNFene metamaterials. (A)** Reflection loss values as a function of frequency of CNF aerogel. (**B**) Reflection loss values as a function of frequency of CNFene. (**C**) Reflection loss values as a function of frequency of CFO-CNFene metamaterial.
